# The German Ethical Culture Scale (GECS): Development and First Construct Testing

**DOI:** 10.3389/fpsyg.2019.01667

**Published:** 2019-07-16

**Authors:** Carmen Tanner, Katharina Gangl, Nicole Witt

**Affiliations:** ^1^Leadership Excellence Institute Zeppelin, Zeppelin University, Friedrichshafen, Germany; ^2^Department of Banking and Finance, University of Zurich, Zurich, Switzerland; ^3^Department of Economic and Social Psychology, University of Göttingen, Göttingen, Germany

**Keywords:** ethical culture measure, compliance-based culture, integrity-based culture, ethics management, organizational culture

## Abstract

Misconduct in organizations (such as fraud, stealing, deception, and harming others) is not only a matter of some “bad apples” but also related to the organizational context (“bad barrels”), which can facilitate either ethical or unethical behaviors. Given the financial crisis and recurring corporate ethics scandals, policymakers, regulators and organizations are interested in how to change their organizational cultures to enhance ethical behavior and to prevent further disasters. For this purpose, organizations need to better understand what strategies and factors of the organizational environment can affect (un)ethical behavior. However, to assess the corporate ethical culture, solid measures are required. Since there is an urgent need to have a German measure to promote research in German-speaking countries, this research developed and tested the German Ethical Culture Scale (GECS). Drawing on a prominent approach that has received much attention from scholars and practitioners alike, the GECS attempts to integrate the notion of compliance- and integrity-based ethics programs (with its focus on how to steer organizations) with the notion of ethical culture (with its focus on what factors inhibit or foster ethical behavior). Three studies with heterogeneous samples of German and Swiss employees and managers were conducted to develop, test and validate the multidimensional scale (total *N* > 2000). Overall, the studies provide first evidence of the measure’s construct, criteria-related and incremental validity. The paper concludes with a discussion of the strengths and weaknesses of the GECS and implications for future research.

## Introduction

The financial crisis and recurring corporate ethics scandals have strengthened the interest of policymakers, regulators and industry in leveraging corporate culture to enhance ethical behavior. There is broad consensus that misconduct in organizations (such as fraud, stealing, deception, and harming others) is not only a matter of some “bad apples” but also related to the organizational context (“bad barrels”), which can facilitate either ethical or unethical behaviors. To manage this challenge, organizations need to better understand what strategies and factors that make up the organizational environment influence (un)ethical behavior. For this purpose, solid measures are required that allow assessing those dimensions, help to provide benchmarks and allow reflecting progress in firms’ cultures. The goal of our research is to develop and provide a first testing of a German measure of features of a corporate ethical culture. While most research in this field has mainly been conducted drawing on English measures, a German measure on ethical culture is still lacking. There is an urgent need to develop a German measure to make it possible to advance research on ethical culture in countries with German-speaking populations, such as Germany, Switzerland, and Austria, which encompass an overall population of approximately 100 million people. However, it is about not only the possibility of advancing research in German-speaking countries but also offering organizations in those countries instruments to obtain information about the existing organizational environment in the firm, to deduce interventions and to monitor potential progress. Not all firms are international, so it cannot be expected that all people understand and speak English fluently.

Conceptually, over the past decades, three main research lines have emerged that examine the ethical environment of an organization: A first stream is related to the notion of *ethical climate.* Most research in this field has referred to the seminal work by Victor and Cullen (1987, 1988). According to them, ethical climate refers to shared perceptions about “what constitutes right behavior” in an organization, thus delivering guidelines for the employees on how they *should* behave (Martin and Cullen, 2006, p. 177). In their pioneering work, Victor and Cullen also developed the Ethical Climate Questionnaire (ECQ), which was originally designed to assess nine various types of ethical climates (Victor and Cullen, 1987, 1988). Although this measure has been criticized for its inability to provide a consistent factor structure, most research has used the ECQ. This research has often found that an employee’s perception of the organization’s focus is associated with unethical behavior and the employee’s attitudes toward the organization (for a review, see Kish-Gephart et al., 2010; Mayer, 2014).

A parallel stream is related to the notion of *ethical culture*. In contrast to ethical climate, ethical culture focuses more on the perceived formal and informal elements of an organizational context that may be likely to encourage ethical conduct (Treviño et al., 1998; Kaptein, 2008a; Ruiz-Palomino and Martínez-Cañas, 2014). As was true for ethical climate, research on ethical culture has consistently found that the perception of an ethical culture among employees is, e.g., associated with more-favorable job attitudes, ethical intentions and behavior (for a review, see Mayer, 2014). In comparison to ethical climate, we believe that the concept of ethical culture better allows interventions, since it draws more directly on the conditions of (un)ethical behavior. However, as Mayer (2014) pointed out, there is still little consistency in not only the conceptualization but also the measurement of ethical culture. While previous studies treated ethical culture as a one-dimensional construct (Treviño et al., 1998), Kaptein, for instance, conceptualized ethical culture as a multi-dimensional construct. Drawing on a virtue-based theory of business ethics, he developed the Corporate Ethical Virtues questionnaire (CEV; Kaptein, 2008a, 2011), which was designed to assess eight organizational virtues. These virtues are posited to represent organizational conditions that may be likely to stimulate employee ethical conduct.

Finally, there is a third prominent stream targeting corporate ethical culture, which has received much attention from scientific scholars and practitioners alike. Since Paine’s (1994) groundbreaking article on how to manage organizational standards, it has become an integral part of organizational discussions on distinguishing between *compliance* and *integrity*. Yet, the governance strategies associated with a compliance- or integrity-based approach are quite different. The *compliance-oriented approach* (also called the command-and-control approach) is mainly designed to prevent violations through control, detection and threats of punishment for misconduct. The *integrity-oriented* approach (also called the value-oriented or self-regulatory approach), on the other hand, combines a concern for legal issues with the goal of supporting ethically sound behavior through encouraging moral self-governance and responsibility for shared values. In contrast to ethical climate and ethical culture, the notions of compliance- and integrity-oriented programs emphasize how to *steer* organizations toward profitability while taking into account consistency with legal and ethical standards (Verhezen, 2010; Webb, 2012; Wieland et al., 2014). Despite their varying characteristics, both ethics programs represent organizational control systems that aim to encourage rule adherence (Weaver and Treviño, 1999; Weaver et al., 1999; Tyler and Blader, 2005).

There is no doubt that compliance- and integrity-based ethics programs are currently the leading pragmatic approaches to ethics used in the business environment (see e.g., OECD, 2009, for a recognition of this general trend in business, or see Wieland et al., 2014, for acknowledging this trend in Germany). Surprisingly, despite this dominance, it has not been adopted in the (empirical) research on ethical culture. To our knowledge, no sound measure assessing those components has thus far been developed.

The purpose of this paper is to take first steps to address this gap by developing a new measure. We believe that there are several reasons that it is necessary to expand previous scales. (1) The prior ethical climate/culture approaches do not say a great deal about how to embed ethics in organizations and managerial practice. Adopting the compliance and integrity framework in a new measure thus complements prior work by emphasizing more how to steer an organization. (2) Integrating the compliance and integrity framework into a measure not only better matches trends in practice but also offers an important means to organizations, risk managers, compliance or ethics officers of reflecting current states and progress in their firm. It also allows comparing the utility of both approaches to achieve adherence to rules and ethical standards. (3) A new measure is also needed to be able to conduct rigorous empirical research on the antecedents, consequences and effectiveness of the applied governance strategies. Astonishingly, empirical studies in this domain are thus far nearly absent. Most inferences for organizations are, as far as we can see, mainly based on theory and cased-based studies (e.g., Paine, 1994; Verhezen, 2010). As rare exceptions, we mention Treviño et al. (1999), Weaver and Treviño (1999), and Tyler and Blader (2005), who have provided first empirical evidence that integrity-based programs are likely to make a unique contribution to predicting unethical behavior and employee attitudes compared to programs that are solely based on command and control. However, our knowledge about what those various programs actually accomplish and how they affect employee’s attitudes and ethical behavior is still severely limited. Thus, developing a measure integrating this framework offers opportunities to examine more thoroughly possible antecedents and consequences of a compliance- and integrity-based culture and the interplay of those programs. (4) A further reason we decided to start developing a new measure was to better account for essential findings from the field of behavioral ethics and social psychology. In particular, behavioral ethics research has, in recent years, provided many important insights about organizational factors that can affect ethical behavior but which have, as far as we can see, not been considered in prior ethical culture measures.

Overall, we aim to develop a new scale that adopts both a compliance and integrity orientation, representing two steering strategies to implement ethics programs, and adopting features of the organizational culture, which can inhibit or facilitate the effectiveness of those governance strategies. Specifically, we propose that to manage ethics, compliance- and integrity-based programs should be supplemented by knowledge about features of the organizational culture, which can inhibit or facilitate the effectiveness of the governance strategies.

## Adopting the Compliance and Integrity Framework

As mentioned above, compliance- or integrity-based programs focus on different strategies for how to *steer* the organization. However, while both compliance- and integrity-based approaches share the same goal (behavior in accordance with organizational rules and values), the procedures to steer the organization are quite different. Whereas the former emphasizes regulation through lawful rules, the latter emphasizes regulation through values (Paine, 1994; Weaver and Treviño, 1999; Verhezen, 2010; Webb, 2012; Wieland et al., 2014). Ethics policies, however, whatever aim they have, have to rely on an effective implementation. Obviously, there may be key features of the organizational environment that can affect the effectiveness of the policies. For example, an organization may attempt to build on shared values such as honesty, fairness and respect; however, the leaders in the firm may not “live” those values, thereby potentially undermining the effectiveness and credibility of the ethics program.

To implement effective ethics programs, board and managers need therefore to know how members of an organization perceive and evaluate the firm’s policies and activities; however, it is also important that they have insights into factors of the organizational context that may affect the effectiveness of the firm’s strategies. The goal of the proposed German measure here is to assess both (1) people’s belief about the extent to which an organization relies on compliance and/or integrity governance procedures and (2) people’s perception about the unwritten but lived norms, expectations and behaviors. Consistent with this goal, we define ethical culture as perceptions about the governance strategies and the effectively implemented norms and expectations that are shared by the members of the organization.

As mentioned earlier, we believe that adopting the compliance and integrity distinction will, among other things, identify new research questions that can advance our knowledge about the consequences of ethical culture. This framework points to the possibility that compliance- or integrity-oriented approaches, despite sharing the same goal, may have different implications for employee motivation, attitudes and behavior (see Stimmler and Tanner, 2019, for developing various propositions). For example, whereas compliance factors may likely be associated with controlled motivation, integrity factors may likely foster autonomous motivation (Gagné and Deci, 2005). Indeed, prompted by corporate scandals and the financial crisis, compliance officers and risk managers have mainly implemented compliance-based programs to prevent legal violations (Paine, 1994). Several authors have pointed out the necessity to move beyond an exclusively compliance-based approach (Paine, 1994; Verhezen, 2010; Webb, 2012; Wieland et al., 2014). For example, it is proposed that an integrity-oriented culture may be more advantageous in managing misconduct, since it builds on encouraging people’s intrinsic motivation to follow the organizational standards. This suggestion is intuitively appealing, but it still awaits empirical examination.

We also expand previous measures by including important insights from the fields of behavioral ethics, social psychology and organizational psychology into organizational factors that are likely to be related to ethical behavior. Drawing on this research, our measure will incorporate dimensions such as the role of ill-conceived goals, accountability or pressure to compromise (which will be detailed below). These dimensions have thus far not been addressed in prior ethical culture measures. With rule viability and rule defectiveness (which will also be detailed below), we will adopt two new dimensions. We learned from discussions with practitioners about those dimensions allegedly representing two essential challenges of compliance management. We continue by describing the German Ethical Culture Scale (GECS).

## Building Blocks of the Gecs

As is common in research on ethical climate or culture, we measure employee perception of both formal elements (e.g., code of ethics or availability of a hotline) and informal ones, since their perception is the reality based upon employee reactions. Building on the converging features of previous measures, we also conceptualize ethical culture as a multi-dimensional concept. The focus will be on dimensions that are proposed to match with a compliance- or integrity-based strategy. As mentioned earlier, the GECS will be designed to assess (1) people’s belief about the extent to which an organization relies on compliance and integrity governance strategies and (2) people’s perception about the lived norms and expectations. These factors might inhibit or promote the implementation of the strategies.

### Compliance Factors

A compliance-based approach to ethics management focuses on preventing misconduct through control, monitoring and punishment (Paine, 1994; Weaver and Treviño, 1999). Such procedures are based on classic economic theory that following rules is a function of extrinsic costs and benefits associated with questionable behavior (e.g., Sutherland, 1983; Tyler and Blader, 2005). Such strategies are also implicitly based on the assumption that people cannot be trusted and are ethically incompetent, which is why external controls are inevitably to prevent wrongdoing (Weaver and Treviño, 1999; Verhezen, 2010; Webb, 2012). Building on past studies and converging elements of previous measures, we propose the following five aspects to reflect a compliance-based culture:

(1) *Controlling* and (2) *Sanctioning* reflect classic governance procedures clearly associated with a compliance strategy. Controlling is the degree to which people believe that they are being monitored and that misconduct is likely to be detected. Sanctioning refers to the extent to which people believe that unethical conduct is not tolerated and will be punished. However, for sanctions strategies to work, organizations must also be willing to invest resources into monitoring behavior and make detection of misconduct sufficiently likely. These two dimensions are similar to previous ethical culture models, which have posited that visibility and punishment of unethical behavior is likely to inhibit unethical behavior (Treviño et al., 1998; Kaptein, 2008a). Empirical studies on the effects of supervision and incentives on unethical behavior are, however, controversial. Some studies suggest that command-and-control strategies do affect undesirable behavior, whereas other studies question the effectiveness of such strategies (see Tyler and Blader, 2005).

Furthermore, we propose three dimensions to reflect the (in)effectiveness of rule-based compliance strategies. (3) *Rule clarity* is the extent to which rules and expectations, as they are often portrayed in codes of ethics or conduct, are sufficiently clear and concrete to employees. This aspect is consistent with Kaptein (2008a), who posited that ethical standards should be concrete, comprehensible and understandable if employees and managers are expected to follow them. Clear rules help to reduce ambiguity and vagueness of ethical expectations, thereby supporting ethical behavior in positive ways. Other authors also pointed out that, for rules to become effective for influencing behavior, one important precondition is that they are accessible, clearly communicated and easy to understand (Schwartz, 2001; Stevens, 2008).

(4) *Rule defectiveness* is the extent to which employees believe that there are ethical gray areas or other challenging situations where corresponding guidelines are missing. According to Jackson (2000), an issue that has been mainly neglected in prior studies on ethics programs is how organizations deal with “ethical gray areas,” i.e., decisions that do not overly attract ethical condemnation but that nevertheless represent characteristic daily work topics that may be considered as ethical issues (e.g., Should I call in sick to have a day off? Should I do personal business on company time? How should I deal with giving and receiving gifts or ethical dilemmas?). Cross-cultural studies have usually found differences between nations and companies concerning which ethical gray areas are perceived as important and which are adopted in the codes of ethics (see Jackson, 2000). It is plausible to assume that a compliance program’s efficiency in shaping behavior is limited if employees believe that important ethical issues and daily challenges are not appropriately addressed in the proposed rules. To render unethical behavior less likely, employees must believe that they have some guidance when they are faced with important ethical issues.

(5) *Rule viability* refers to the degree to which the company’s rules are perceived to complicate and hinder daily work rather than to provide support. Various authors have expressed concerns that if codes and rules are not deemed relevant and useful for daily work, they are less likely to be accepted and adopted for guiding behavior (Schwartz, 2004; Bageac et al., 2011). An additional aspect of rule viability that has thus far hardly been addressed in prior studies is the issue of being faced with too many rules. This aspect can also minimize the perceived usefulness of rules. In fact, the most prominent response to scandals in business in general and the last financial crisis in particular has been to revise laws and to increase regulation. More rules may increase bureaucratic demands, but they may also confuse and disenable employees, limiting the effectiveness of compliance strategies.

### Integrity Factors

An integrity-based approach to ethics management focuses on promoting ethical behavior through encouraging self-governance and responsibility (Paine, 1994; Weaver and Treviño, 1999). Such programs are based on the view that following rules is a function of an individual’s intrinsic desire to follow organizational rules (Tyler and Blader, 2005). Such strategies are also implicitly based on the assumption that employees can be trusted and are prone to follow ethical values. Hence, integrity-oriented approaches are designed to support ethical aspirations and the identification with and internalization of ethical standards (Weaver and Treviño, 1999; Verhezen, 2010; Webb, 2012). We propose the following aspects to reflect an integrity-based culture:

We deem (6) *accountability* and (7) *leader’s role modeling* to reflect two essential governance procedures that are associated with an integrity strategy. Accountability refers to the extent to which people are clear about who is responsible for which tasks and has to justify one’s actions to others. To our knowledge, accountability has not been addressed in previous ethical cultural models. The argument is, however, that if an organization makes clear that its members are responsible for what they do, this action can intrinsically motivate employees to feel a personal responsibility and a desire to bring behavior in line with corporate rules and ethical standards. We believe that, in contrast, an organization lacking such a governance strategy is more likely to encourage rationalization processes, such as denial of responsibility (Anand et al., 2004).

It is important to acknowledge, however, that accountability has at least three facets. We use the term “task accountability” to refer to who is accountable for which tasks. Being a member of an organization with a hierarchical structure or being involved in teams and collective decision-making tasks is likely to provide people with opportunities to free themselves from personal responsibility. Bandura (1990, 1999) called, e.g., passing the cause of wrongdoing to others displacement of responsibility, and the dispersion of tasks and blame across the members of a group diffusion of responsibility. Consistent with past research in social psychology and behavioral ethics, we refer to “outcome” and “procedural accountability” when organizational members are expected either to justify the results of their decisions (outcome) or to justify how decisions were made (procedural). Empirical studies have shown that accountability can encourage self-critical and deliberate thinking (e.g., Lerner and Tetlock, 1999). The studies by Pitesa and Thau (2013) are also noteworthy. They have provided evidence that organizations holding their employees accountable for their choice procedures (compared to holding them accountable for performance outcomes) reduced agents’ propensity to behave in a self-serving, unethical manner. In other words, employees should in practice be judged on not only the basis of the achieved outcomes but also how those outcomes were accomplished.

Leader’s role modeling refers to the extent to which employees perceive their top management or direct supervisors as role models for ethical conduct. Several authors have proposed ethical leadership to be a crucial element of a value-based organization that guides employee thought and action (see e.g., Treviño et al., 1998; Kaptein, 2008a; Ruiz et al., 2011; Wieland et al., 2014). Leader’s behaviors do reflect the values of the organizational culture. Therefore, managers and supervisors do play a crucial role in setting the ethical tone in an organization and by living up to the values. Furthermore, through a process of social learning (Bandura, 1986), employees are likely to adopt the values and behaviors of the managers.

Many empirical studies have shown that leaders being perceived as ethical models is likely to affect followers’ ethical intentions and behaviors in positive ways (e.g., Brown and Treviño, 2006; Tanner et al., 2010; Ruiz et al., 2011; Ruiz-Palomino and Linuesa-Langreo, 2018). Some authors treat *role modeling of top management* and *role modeling of supervisors* as two distinct categories that affect employees’ responses in distinct ways (Kaptein, 2008a; Ruiz et al., 2011). Both, however, are expected to be negatively related to unethical behavior.

Furthermore, three dimensions are proposed to hinder the effectiveness of integrity-based strategies. (8) *Pressure to compromise* is the extent to which people experience role/value conflicts and pressure from the organization to counter their own sense of right and wrong. The sources of such pressures may be job or role demands, authority figures or working teams that push the employee in behavioral directions that are inconsistent with ethical standards. Previous organizational, business or behavioral ethics research suggests that employees who feel pressure to behave counter to their conscience are likely to suffer from stress and burnout (e.g., Örtqvist and Wincent, 2006; Eatough et al., 2011; Kammeyer-Mueller et al., 2012). A meta-analytic study by Örtqvist and Wincent (2006) indicated that among the most prominent consequences of role conflict were job dissatisfaction, loss of organizational commitment and emotional exhaustion. Another meta-analysis by Eatough et al. (2011) found a negative relationship between role conflict and organizational citizenship behavior (OCB).

It seems therefore plausible to assume that organizational members’ believing that they are expected to compromise their own values is likely to discourage intrinsic motivation to identify with organizational values and to render unethical behavior more likely. Consistent with this point, empirical studies have shown that a mismatch between the expectations of the employees and the organization is likely to not only lower job satisfaction but also increase cheating (Burks and Krupka, 2012) or other forms of unethical behavior (Suar and Khuntia, 2010).

(9) *Obedience* refers to the extent to which people believe that they are expected to be subordinate, keeping authorities and tasks unquestioned (Treviño et al., 1999). Such expectations are likely to enforce a culture of fear and silence rather than a culture of voice (Morrison and Milliken, 2000; Kish-Gephart et al., 2009). While research has suggested multiple factors that can affect the choice to remain silent or to speak up (for an overview, see Morrison, 2014), one common explanation for why people hesitate to speak up is related to fear. Kish-Gephart et al. (2009) found across several studies that a substantial number of respondents reported remaining silent out of fear of experiencing negative personal, social or material consequences. If organization members feel uncomfortable to speak up, such people hardly feel encouraged to practice self-regulation and moral responsibility. In contrast, in such an environment, people may feel not committed and willing to adhere to corporate rules and standards. They may also be likely to withhold input about questionable practices (Snell, 1999; Miceli et al., 2008; Detert et al., 2010). Consistent with this reasoning, Kaptein (2011), for example, found that the opportunity for employees to raise concerns and discuss ethical issues is associated with less-unethical behavior. Additionally, Treviño et al. (1999) found that employees perceiving a structure that expects obedience from them were less willing to report ethical or legal violations or to forward bad news to management.

(10) *Ill-conceived goals* are the extent to which the organization is seen as mainly relying on competitive and economic goals (Bazerman and Tenbrunsel, 2011). Thus far, prior ethical culture models have not addressed this issue. A growing body of research in the field of behavioral ethics, however, has shown that despite the beneficial role of goals in increasing achievement motivation and productivity, goals can also lead to undesirable side effects by encouraging unethical behavior. Indeed, empirical research has demonstrated that people facing competitive or too challenging (also stretch) goals (Barsky, 2008) are more likely to exhibit cheating (e.g., Schweitzer et al., 2004; Schwieren and Weichselbaumer, 2010; Welsh and Ordóñez, 2014) and lower intrinsic work motivation (Ordóñez et al., 2009). Similarly, Van Yperen et al. (2011) have shown that interpersonal achievement goals (that emphasize competition among colleagues) are more likely than are intrapersonal goals (that emphasize mastering a task) to increase cheating. Thus, the argument here is that too challenging, competitive goals hardly support the development of moral responsibility, thereby making unethical behavior more likely.

Furthermore, the effectiveness of integrity-oriented programs may also be undermined by an organization focusing only on economic or self-interested goals in the workplace while not questioning its compatibility with ethical aspirations. For example, an organization may attempt to build on integrity and ethical values, but employees may nevertheless perceive their organizational environment as emphasizing only economic and egoistic goals. Consistent with this point, Kish-Gephart et al. (2010) found meta-analytic evidence for a positive relationship between an egoistic ethical climate and unethical behavior.

Thus, acknowledging the importance of the role of goal-setting, this dimension was also included in our instrument. However, as with the dimension accountability, we distinguish between various facets of ill-conceived goals. Acknowledging this literature, we distinguish between goal-setting with regard to its focus on “competition” and with regard to its focus on “economic” goals. An ethical culture mainly focusing on competition and economic goals is expected to be less likely to promote responsibility and adherence to ethical standards and behavior.

## Overview of Samples and Studies

The primary objective of the following studies was to develop and assess the validity of the GECS. For the following studies, data from three independent and heterogeneous samples of employees and managers in Switzerland and Germany were used. To test the questionnaire, it was important to us to have a broad sample of employees and managers from various economic sectors and from various hierarchical levels. Participants were therefore recruited from panels of participants of market research agencies. All data were collected through online surveys. Samples A and B were used to develop the instrument and to perform an exploratory factor analysis (EFA) and confirmatory factor analyses (CFAs) to provide first evidence for the construct validity of the measure. Sample B was also used to assess the criterion related and incremental validity by testing its relation to (observed) deviant workplace behaviors, based on regression analyses. Finally, Sample C was used to take first steps to provide evidence of the convergent validity of the GECS. The studies were conducted in accordance with the ethical standards of the Zeppelin University and the Swiss Psychological Society. The samples are briefly described below. Table 1 reports further work-related characteristics.

**TABLE 1:** Further work-related descriptive statistics of Samples A, B, and C.

	**Sample A (*N* = 300)**	**Sample B (*N* = 990)**	**Sample C (*N* = 991)**

**Country**	**Switzerland**	**Germany**	**Switzerland/Germany**
**Employment**			
Full-time	91.7%	72.9%	66.7%
Part-time	5.7%	27.1%	33.3%
**Job position**			
Non-Management Level	54.3%	58.4%	65.9%
Lower management	17.0%	17.8%	15.0%
Middle management	13.7%	17.7%	12.7%
Upper management	15.0%	6.2%	6.3%
**Tenure (in current organization)**			
<1 year	9.3%	0.3%	1.1%
1–2 years	16.3%	15.8%	25.6%
3–5 years	21.0%	22.3%	21.1%
6–10 years	19.7%	20.4%	18.5%
>10 years	33.7%	41.2%	33.6%
**Company size**			
<50 employees	32.0%	28.1%	29.4%
50–249 employees	23.3%	20.2%	22.5%
250–10,000 employees	32.7%	36.8%	36.7%
>10,000 employees	12.0%	14.9%	11.4%
**Economic Sector**			
Agriculture and Energy	3.7%	2.0%	1.3%
Industry	28.0%	18.5%	17.3%
Services	57.7%	71.1%	73.3%
Others	10.7%	8.4%	8.1%

*In sample A, 2.6% of participants were currently unemployed but worked in an organization in the past 2 years for at least 9 months.*

*Sample A:* This sample consisted of 300 (German-speaking) Swiss employees and managers working in divers economic sectors. Of these respondents, 36.3% were female, and mean age was 42.6 years (*SD* = 12.6, range: 16–72 years).

*Sample B:* This sample consisted of 990 German employees and managers, after 17 participants had to be excluded from the analysis because of a lack of variation in their answering pattern. Of these respondents, 46.6% were female, and mean age was 44.6 years (*SD* = 11.23, range: 21–74 years).

*Sample C:* This sample consisted of 493 German and 498 Swiss participants (total *N* = 991), after exclusion of nine participants due to a lack of variation in their answering pattern. Of this sample, 46.1% were female, and mean age was 44.4 years (*SD* = 12.81, age range: 16–75 years).

In the following, we present the development of the GECS, its factorial structure and psychometric properties (Study 1a, 1b). We then present results on the criterion-related and incremental validity (Study 2) and tests on construct validity with external constructs (Study 3).

### Study 1a – Initial Development of Instrument

In a first preliminary step, an initial pool of items was generated based on the definition of the dimensions proposed to be related to compliance and integrity, as explained in the previous section. We first created items to assess the following constructs: controlling, sanctioning, rule clarity, rule defectiveness, and rule viability (proposed to represent compliance factors) and accountability, role modeling of top management, role modeling of supervisors, pressure to compromise, obedience, and ill-conceived goals (proposed to represent integrity factors).

Our aim was to develop simple items that avoid, as often as possible, terms such as “ethical” or “moral.” Examination of previous instruments revealed that they often include items that may be somewhat precarious, since they utilize the word “ethical” in the wording. Such items, however, may be problematic since they require from the respondents an understanding of what characterizes ethical or responsible conduct, which is often not self-evident. We tried to avoid this shortcoming whenever possible for this reason and also as an attempt to minimize socially desirable responses.

Furthermore, our intention was ultimately to have a relatively short but sound inventory. Of course, the generally accepted argument is that longer instruments tend to have better psychometric properties than shorter ones do (Gosling et al., 2003). However, researchers are often faced with the problem that time is limited and employees or managers are simply not willing to fill out lengthy questionnaires. In such cases (as is true in our research domain), practicable and efficient measures are necessary. We therefore sought to have a minimum of three items for each subscale. For accountability and ill-conceived goals, since they contain nuanced facets, we wished to have a minimum of six items (accountability with its facets task, outcome and procedural accountability) and four items (ill-conceived goals with its facets competition and economic goals).

To this end, 34 items were developed by us, and 27 items were adapted from prior instruments (e.g., Treviño et al., 1998; Brown et al., 2005; Kaptein, 2008a). The items were then discussed with three doctoral candidates experienced with the field. Based on their inputs, items were reworded. An initial pool of 61 items was then subjected to a first EFA (principle axis factor analysis, promax rotation) with data collected from Sample A (*N* = 300). Participants rated each item using a 5-point Likert-style format 1 (*strongly disagree*) to 5 (*strongly agree*), including the response option *no answer is applicable*.

Based on this EFA, we removed 23 items with loadings lower than 0.45 on one factor and cross-loadings higher than loadings of 0.30. Rerunning the EFA with the remaining 38 items resulted in nine factors (eigenvalues > 1). However, recognizing that several subscales were insufficiently represented, we repeated the item-generation and item-selection process in Study 1B.

### Study 1b – Instrument Refinement and Factor Analyses

We conducted a second iteration of instrument development. Using EFA, we examined the scale’s factorial structure and psychometric properties with data collected from Sample B (*N* = 990). The goal of Study 1B was also to assess the robustness of the proposed factor structure by conducting CFAs across Sample B and Sample C (*N* = 991).

#### Measure

In addition to the remaining items from Study 1a (38 items), new items were created in this second iteration. We added more items to those constructs, which were suggested to have nuanced facets (accountability, ill-conceived goals) (plus 15 items), and we also created some new items in the remaining scales (4 items) just to be on the safe side when subjecting the items to new analyses. In this stage, we also discussed content and comprehensiveness of the items with three business ethics scholars and three specialists with significant practical expertise in ethics management. Especially from the practitioners, we learned about rule viability and rule defectiveness as representing two essential challenges in the managerial practice of compliance management, which we consequently also included in our item pool (plus 10 items). Based on the input of those external experts, items were adapted, resulting in a final pool of 67 items.

The items describe concepts related to compliance (controlling, sanctioning, rule clarity, rule defectiveness, and rule viability) and integrity (accountability, role modeling of top management, role modeling of direct supervisor, pressure to compromise, obedience, and ill-conceived goals). Again, participants rated each item on a 5-point scale (1 = *strongly disagree*, 5 = *strongly agree*), including the response option *no answer is applicable*. The items of the final instrument (37 items) – the original German items and the English translation (based on Brislin, 1976) – can be found in the Supplementary Table 1. Sample items include the following (English translation): “In my work environment, measures are carried out to detect rule violations and misconduct” (controlling); “In my work environment, people who engage in dishonest behavior are disciplined” (sanction); “My organization makes it sufficiently clear to me which behaviors are right or wrong” (rule clarity); “Some of the organization’s rules are intentionally not or vaguely defined” (rule defectiveness); “The organization’s code of conduct makes everything complicated” (rule viability); “In my workplace, how goals are achieved also plays a role” (accountability); “My direct supervisor is a good model of integrity” (role modeling of direct supervisor); “In my work environment, I am sometimes asked to do things that are in conflict with my conscience” (pressure to compromise); “I am expected to do what I am told” (obedience); and “In my workplace, I can only make a career by outperforming others” (ill-conceived goals).

#### Results

Building on the data from Sample B, the initial item analyses revealed no peculiarities. The pool of 67 items was then again subjected to an EFA (principal component analysis, promax rotation).

We removed items with factor loadings less than 0.45 on one factor and cross-loadings higher than loadings of 0.30. These changes ultimately resulted in an instrument of 37 items (see Supplementary Table 1). Rerunning the EFA on this instrument reduced the 37-item measures to 10 distinct factors (eigenvalues > 1.0) corresponding to controlling (three items), sanctioning (three items), rule clarity (three items), rule defectiveness (three items), rule viability (three items), accountability (six items), pressure to compromise (four items), obedience (three items), and ill-conceived goals (four items). Role modeling of top management and role modeling of direct supervisors were not revealed as two separate factors. They were therefore combined into one leader’s role modeling factor (five items). All factors accounted for 72.07% of the variance. The factor loadings are reported in Table 2.

**TABLE 2:** EFA factor loadings and CFA standardized factor loadings (Study 1b).

**Factor loadings**												

	**PCA**										**CFA**	
	**(Sample B)**										**(Sample B and C)**	
**Item**	**1**	**2**	**3**	**4**	**5**	**6**	**7**	**8**	**9**	**10**	***λ _B_***	***λ_C_***
Controlling 1	0.09	0.02	–0.01	–0.02	–0.05	–0.07	0.07	–0.07	–0.08	**0.92**	**0.81**	**0.80**
Controlling 2	–0.01	–0.06	0.02	0.04	–0.06	–0.03	0.06	–0.04	0.13	**0.91**	**0.85**	**0.85**
Controlling 3	–0.10	0.10	–0.02	–0.03	0.22	0.16	–0.20	0.27	–0.08	**0.48**	**0.48**	**0.59**
Sanctioning 1	–0.05	0.05	0.00	–0.03	–0.07	–0.03	0.04	**0.94**	–0.07	–0.02	**0.78**	**0.80**
Sanctioning 2	0.01	–0.07	–0.04	–0.01	–0.06	0.01	0.01	**0.89**	0.08	0.01	**0.83**	**0.73**
Sanctioning 3	0.19	–0.15	–0.02	0.17	0.05	–0.05	0.06	**0.54**	0.10	–0.08	**0.55**	**0.58**
Rule Clarity 1	0.00	0.03	–0.01	**0.92**	0.01	–0.01	0.00	0.05	0.01	–0.03	**0.91**	**0.88**
Rule Clarity 2	–0.05	0.05	0.03	**0.89**	–0.03	–0.03	–0.01	0.01	0.00	0.02	**0.83**	**0.88**
Rule Clarity 3	0.06	–0.07	–0.01	**0.89**	0.00	0.00	0.04	–0.05	0.02	0.02	**0.86**	**0.82**
Rule Defectiveness 1	–0.08	–0.04	0.02	–0.02	0.01	0.02	**0.85**	0.00	0.07	0.04	**0.83**	**0.79**
Rule Defectiveness 2	–0.05	0.03	0.01	0.04	0.04	0.09	**0.82**	–0.02	–0.03	0.03	**0.88**	**0.88**
Rule Defectiveness 3	0.02	0.18	–0.01	0.02	0.07	–0.04	**0.78**	0.10	–0.10	0.01	**0.73**	**0.70**
Rule Viability 1	0.10	–0.09	0.00	0.01	–0.05	**0.96**	–0.04	–0.01	0.02	0.03	**0.72**	**0.69**
Rule Viability 2	0.07	0.06	0.04	–0.04	–0.03	**0.87**	0.05	0.02	0.02	–0.03	**0.88**	**0.90**
Rule Viability 3	0.04	0.02	0.00	–0.02	–0.07	**0.81**	0.09	–0.05	0.09	–0.06	**0.87**	**0.82**
Accountability 1	–0.13	–0.07	**0.87**	–0.02	0.10	0.11	–0.05	–0.02	–0.12	–0.02	**0.67**	**0.82**
Accountability 2	–0.01	–0.06	**0.83**	–0.05	0.02	0.06	0.07	–0.01	0.10	–0.01	**0.64**	**0.74**
Accountability 3	0.10	0.09	**0.74**	0.02	–0.16	–0.10	0.08	–0.12	–0.01	–0.02	**0.68**	**0.74**
Accountability 4	0.11	–0.05	**0.66**	0.01	0.02	–0.14	–0.02	–0.01	0.07	–0.03	**0.74**	**0.77**
Accountability 5	–0.04	–0.04	**0.60**	0.08	0.09	0.16	0.05	0.13	–0.13	0.07	**0.61**	**0.55**
Accountability 6	0.11	0.07	**0.56**	0.05	–0.05	–0.12	–0.15	0.00	0.16	0.05	**0.66**	**0.64**
Role Modeling 1	**0.96**	0.00	–0.03	0.02	0.05	0.09	–0.04	–0.06	0.00	0.03	**0.94**	**0.92**
Role Modeling 2	**0.95**	0.00	–0.06	0.08	0.03	0.11	–0.02	–0.08	–0.05	0.02	**0.90**	**0.86**
Role Modeling 3	**0.94**	–0.01	–0.05	0.04	0.09	0.10	–0.05	–0.02	0.00	0.01	**0.89**	**0.91**
Role Modeling 4	**0.68**	0.09	0.16	–0.13	0.02	–0.10	–0.03	0.14	–0.04	–0.01	**0.67**	**0.68**
Role Modeling 5	**0.65**	0.10	0.16	–0.09	–0.10	–0.03	0.03	0.15	–0.02	–0.01	**0.62**	**0.67**
Pressure To Compromise 1	0.04	**0.92**	–0.02	0.02	–0.07	–0.02	0.02	–0.03	0.06	0.03	**0.87**	**0.87**
Pressure To Compromise 2	0.07	**0.89**	–0.05	0.04	0.01	–0.02	0.01	–0.02	0.06	–0.01	**0.86**	**0.85**
Pressure To Compromise 3	0.05	**0.86**	–0.04	–0.02	0.00	–0.04	0.03	–0.05	0.02	0.02	**0.81**	**0.76**
Pressure To Compromise 4	–0.04	**0.81**	0.04	–0.05	0.02	0.03	0.08	0.03	0.00	–0.05	**0.87**	**0.87**
Obedience 1	0.03	0.01	0.00	0.04	–0.01	0.05	–0.03	0.01	**0.89**	0.00	**0.77**	**0.81**
Obedience 2	–0.10	0.10	0.02	0.03	0.05	0.02	0.00	0.02	**0.80**	0.01	**0.90**	**0.91**
Obedience 3	–0.13	0.17	–0.01	–0.09	0.13	0.08	–0.04	0.04	**0.54**	0.02	**0.72**	**0.84**
Ill-Conceived Goals 1	0.01	–0.20	0.02	–0.04	**0.86**	–0.06	0.07	–0.07	0.14	0.04	**0.63**	**0.63**
Ill-Conceived Goals 2	0.26	–0.07	–0.05	–0.08	**0.85**	–0.17	0.14	–0.03	0.07	–0.03	**0.56**	**0.53**
Ill-Conceived Goals 3	–0.09	0.16	0.01	0.05	**0.66**	0.07	0.01	–0.01	–0.10	–0.05	**0.76**	**0.85**
Ill-Conceived Goals 4	–0.11	0.27	0.08	0.11	**0.65**	0.05	–0.14	–0.02	–0.11	–0.04	**0.76**	**0.85**

*Factors: 1 = Leader’s Role Modeling, 2 = Pressure to Compromise, 3 = Accountability, 4 = Rule Clarity, 5 = Ill-Conceived Goals, 6 = Rule Viability, 7 = Rule Defectiveness, 8 = Sanctioning, 9 = Obedience, 10 = Controlling; λ = standardized factor loadings of the CFA (Model C with 10 first-order factors and correlated error terms for role modeling of top management items).*

Table 3 reports the means, standard deviations, internal consistencies (Cronbach’s alphas), and intercorrelations among the subscales. The subscales in this table were calculated by averaging the corresponding item scores. As shown, each scale showed good internal consistency (αs between 0.72 and 0.91).

**TABLE 3:** Correlation matrix of the GECS factors, means, standard deviations, and Cronbach’s alphas (Sample B, Study 1b).

	***M* (*SD*)**	**α**	**1**	**2**	**3**	**4**	**5**	**6**	**7**	**8**	**9**
(1) Controlling	3.43 (0.90)	0.72									
(2) Sanctioning	3.47 (0.88)	0.74	0.46^∗∗∗^								
(3) Rule clarity	3.99 (0.91)	0.90	0.39^∗∗∗^	0.43^∗∗∗^							
(4) Rule defectiveness	2.71 (1.08)	0.85	–0.04	–0.13^∗∗∗^	–0.23^∗∗∗^						
(5) Rule viability	2.73 (1.08)	0.86	–0.01	–0.14^∗∗∗^	–0.16^∗∗∗^	0.49^∗∗∗^					
(6) Accountability	3.59 (0.73)	0.83	0.36^∗∗∗^	0.48^∗∗∗^	0.44^∗∗∗^	–0.23^∗∗∗^	–0.27^∗∗∗^				
(7) Leader’s role modeling	3.43 (1.01)	0.91	0.28^∗∗∗^	0.43^∗∗∗^	0.36^∗∗∗^	–0.31^∗∗∗^	–0.29^∗∗∗^	0.59^∗∗∗^			
(8) Pressure to compromise	2.38 (1.09)	0.91	–0.01	–0.11^∗∗∗^	–0.17^∗∗∗^	0.57^∗∗∗^	0.49^∗∗∗^	–0.20^∗∗∗^	–0.34^∗∗∗^		
(9) Obedience	2.95 (1.00)	0.83	0.02	–0.06	–0.09^∗∗^	0.39^∗∗∗^	0.42^∗∗∗^	–0.21^∗∗∗^	–0.39^∗∗∗^	0.59^∗∗∗^	
(10) Ill-conceived goals	2.81 (0.91)	0.77	0.19^∗∗∗^	0.09^∗∗^	0.03	0.36^∗∗∗^	0.34^∗∗∗^	0.00	–0.12^∗∗∗^	0.51^∗∗∗^	0.46^∗∗∗^

*^∗∗^*p* < 0.01, ^∗∗∗^*p* < 0.001.*

The GECS with the final item set (37 items) was then sent online to Sample C. We next tested robustness of the proposed model by conducting a series of CFAs across Samples B and C. CFAs were performed using AMOS 22 maximum likelihood estimation of covariance. The answer *no answer is applicable* was coded as missing values; hence, we were allowed to estimate means and intercepts.

Five models were estimated and compared: Model A with one factor, Model B with two factors (compliance and integrity) and Model C with the proposed 10 factors. With Models D and E, we also examined whether the 10 factors can be summarized into higher-order factors. In the theory section, we proposed that compliance and integrity would converge at two higher-order constructs (controlling, sanctioning, clarity, defectiveness and viability of rules should load onto a compliance factor; accountability, leader’s role modeling, pressure to compromise, obedience, ill-conceived goals should load onto an integrity factor, Model E). We compared this Model E with the alternative Model D (10 factors loading on one higher-order construct).

To assess the model fit, the chi-square (*χ2/df*), comparative fit index (CFI), Tucker-Lewis-Index (TLI) and root mean square error of approximation (RMSEA) were used. According to previous studies, it is suggested that *χ2/df* should be equal to or less than 3 or at least less than 5. For CFI and TLI, values above 0.90 are regarded as acceptable, and values close to or greater than 0.95 are regarded as good fit. For RMSEA, values equal to or less than 0.07 indicate a good fit. To compare the models, we used the Akaike Information Criterion (AIC), with smaller values indicating a better-fitting model (see, e.g., Wheaton et al., 1977; Hooper et al., 2008).

An examination of modification indices revealed high error covariance between the two top management role-modeling items in Models C, D, and E. When the two error terms were free to correlate, the fit was clearly improved. Importantly, this modification also makes theoretical sense because prior research suggested that role modeling of top management and role modeling of supervisors are two distinct constructs (Kaptein, 2008a; Ruiz et al., 2011).

Table 4 displays the fit statistics of the models. The results repeatedly revealed across all samples that the ten-factor solution (Model C) fit the data better than did the one- or two-factor solution (Models A and B), which was also confirmed by *χ*2-difference tests between the three nested first-order models. Based on the criteria, all fit indices indicated a good or acceptable fit for the 10-factor model. The same applies to the two second-order models (D and E), with the exception of CFI and TLI, which were marginally below the recommended criteria. Comparing the two second-order factor models, *χ*2-difference tests pointed to Model E (with two second-order factors: Integrity and Compliance) fitting the data better than did Model D (with one second-order factor: Ethical Culture). Testing the robustness of our Models C, D, and E separately for the Swiss and German respondents of Sample C also yielded very similar results for both countries. Overall, the results largely supported the 10-factor solution but also deemed second-order factors acceptable. CFA standardized factor loadings of the 10-factor model (Model C) are reported in Table 2.

**TABLE 4:** Goodness-of-Fit Indices of CFA models (Study 1b).

**Model**	**χ^2^(*df*)**	**χ^2^/*df***	**CFI**	**TLI**	**RMSEA**	**AIC**	**χ^2^ difference tests**		
								**Δχ^2^**	**Δ*df***
*Sample B (N* = 990)									
A: 1 factor (Ethical Culture)	13736.22 (629)^∗∗∗^	21.84	0.39	0.36	0.15	13958.22	A–C	11897.49^∗∗∗^	46
B: 2 factors (Integrity, Compliance)	12485.15 (628)^∗∗∗^	19.88	0.43	0.36	0.14	12709.15	B–C	10646.42^∗∗∗^	45
C: 10 factors^a^	1838.73 (583)^∗∗∗^	3.15	0.94	0.93	0.05	2152.73			
D: 10 first order^a^, 1 second-order factor (EC)	3015.02 (618)^∗∗∗^	4.88	0.89	0.87	0.06	3259.02	D–E	32.58^∗∗∗^	1
E: 10 first order^a^, 2 second-order factors	2982.44 (617)^∗∗∗^	4.83	0.89	0.87	0.06	3228.44			
(Integrity, Compliance)									
*Sample C (N =* 991*)*									
A: 1 factor (Ethical Culture)	13025.28 (629)^∗∗∗^	20.71	0.42	0.35	0.14	13247.28	A–C	11153.60^∗∗∗^	46
B: 2 factors (Integrity, Compliance)	11896.09 (628)^∗∗∗^	18.94	0.47	0.41	0.14	12120.09	B–C	10024.41^∗∗∗^	45
C: 10 factors^a^	1871.68 (583)^∗∗∗^	3.21	0.94	0.93	0.05	2185.68			
D: 10 first order^a^, 1 second-order factor (EC)	3001.54 (618)^∗∗∗^	4.86	0.89	0.87	0.06	3245.54	D–E	7.07^∗∗^	1
E: 10 first order^a^, 2 second-order factors	2994.47 (617)^∗∗∗^	4.85	0.89	0.87	0.06	3240.47			
(Integrity, Compliance)									

*EC, Ethical Culture. In models C, D, and E, the error terms for the two-role modeling of top management items were allowed to correlate. ^*a*^The 10 factors are as follows: controlling, sanctioning, rule clarity, rule defectiveness, rule viability, accountability, leader’s role modeling, pressure to compromise, obedience, ill-conceived goals. *^∗∗^p* < 0.01; *^∗∗∗^p* < 0.001.*

We examined convergent validity of the 10 factors by assessing average variance extracted (AVE) in the CFAs (Fornell and Larcker, 1981; Hair et al., 2014). The results are reported in Table 5. With regard to AVE, the analyses revealed that most constructs were greater than 0.50, as recommended (Fornell and Larcker, 1981). Just for accountability (AVE = 0.45) and ill-conceived goals (AVE = 0.46), but only in Sample B, the criterion was not met. We assume that the reason the AVE of those factors is below the recommended threshold is that accountability and ill-conceived goals may be more heterogeneous in content. However, with data from Sample C, the criterion of 0.5 was met for all constructs.

**TABLE 5:** Convergent and discriminant validity of the 10-Factor Model (Model C) (Study 1b).

**Latent constructs**	**AVE**	**Square root of AVE**	**Latent construct correlation**								
			**1**	**2**	**3**	**4**	**5**	**6**	**7**	**8**	**9**
*Sample B (N = 990)*											
(1) Controlling	0.54	0.73									
(2) Sanctioning	0.53	0.73	0.52								
(3) Rule clarity	0.75	0.87	0.44	0.48							
(4) Rule defectiveness	0.66	0.81	–0.11	–0.16	–0.27						
(5) Rule viability	0.68	0.83	–0.10	–0.15	–0.19	0.58					
(6) Accountability	0.45	0.67	0.44	0.54	0.50	–0.29	–0.35				
(7) Leader’s role modeling	0.66	0.81	0.33	0.41	0.38	–0.34	–0.29	0.63			
(8) Pressure to compromise	0.73	0.85	–0.09	–0.10	–0.18	0.63	0.57	–0.24	–0.36		
(9) Obedience	0.64	0.80	–0.02	–0.04	–0.07	0.43	0.48	–0.23	–0.40	0.64	
(10) Ill-conceived goals	0.46	0.68	0.10	0.11	0.05	0.43	0.43	–0.03	–0.17	0.62	0.55
*Sample C (N = 991)*											
(1) Controlling	0.56	0.75									
(2) Sanctioning	0.50	0.71	0.62								
(3) Rule clarity	0.74	0.86	0.50	0.58							
(4) Rule defectiveness	0.63	0.79	–0.06	–0.17	–0.30						
(5) Rule viability	0.65	0.81	0.08	–0.07	–0.14	0.48					
(6) Accountability	0.51	0.71	0.33	0.47	0.48	–0.39	–0.39				
(7) Leader’s role modeling	0.67	0.82	0.30	0.36	0.40	–0.37	–0.34	0.63			
(8) Pressure to compromise	0.70	0.84	0.01	–0.17	–0.23	0.59	0.52	–0.39	–0.47		
(9) Obedience	0.72	0.85	0.07	–0.07	–0.05	0.35	0.43	–0.35	–0.40	0.57	
(10) Ill-conceived goals	0.53	0.73	0.20	0.13	0.03	0.33	0.43	–0.20	–0.27	0.53	0.50

*AVE, average variance extracted.*

We also examined divergent validity by AVE. Adequate divergent validity is given when the square root of the variance extracted from each of the factors is greater than the correlations between each pair of factors (Fornell and Larcker, 1981). As shown in Table 5, all factors satisfy this condition with data from both Samples B and C.

### Study 2 – Criterion-Related and Incremental Validity

To establish criterion-related validity, the association between the GECS and deviant workplace behavior was considered with data from Sample B. To minimize potential social desirability bias, we administered *observed* and not self-reported deviant workplace behavior (which is assumed to be more susceptible to socially desirable responding and impression management), and we used different response formats to assess the predictor and criterion variables. Specifically, we expected controlling, sanctioning, rule clarity, accountability, and leader’s role modeling to be associated with less observed deviant workplace behavior. We expected rule defectiveness, rule viability, pressure to compromise, obedience, and ill-conceived goals to be associated with more observed misconduct.

Several authors have argued that to align employee’s behavior with ethical standards, it is important to move beyond compliance-oriented strategies, since control and punishment may prevent wrongdoing, but they do not necessarily change convictions (e.g., Paine, 1994; Tyler and Blader, 2005). Integrity-based strategies, on the other hand, encourage personal commitment to ethical aspirations, thereby being more successful in affecting behavior (e.g., Weaver and Treviño, 1999). If so, we should be able to find that integrity factors will account for variance increments over and above the compliance factors.

#### Measures

##### Observed deviant workplace behavior

Beyond the GECS, participants were also asked to report observed misconduct in the workplace. Drawing on a German version of Kaptein’s (2008b) scale of observer-reports of unethical behavior (Kaptein, 2011; Zuber and Kaptein, 2014), participants were asked how often in the past 12 months they had observed or had first-hand knowledge of intra- or extra-organizational deviant behaviors in their work environment (e.g., discriminating against others due to age, gender, or sexual orientation; violating contracts or lawful rules; misleading others; or stealing; 19 items). Consistent with Kaptein (2008b, 2011) and Zuber and Kaptein (2014), responses were given on a 5-point frequency scale (*never*, *rarely, sometimes, often, almost always)*, including the response option *no answer is applicable*.

##### Formal factors of ethics programs

Participants were also asked about the availability of formal codes or other forms of internal formal ethics programs in their organization, such as the existence of a code of ethics, ethical training, a telephone hotline, availability of ethics officers, formal ethics controls or an ethics report (adapted from Kaptein, 2014). Possible answers were *yes*, *no*, and *not applicable*.

#### Results

To assess criterion-related and incremental validity, correlation and regression analyses were conducted to examine the relation between the GECS dimensions and the dependent variable (observed deviant workplace behavior). As mentioned above, the observer-based reports on deviant workplace behaviors were assessed using a 5-point frequency scale. Though such scales are widely used in social sciences, we decided to dichotomize the response scale, partly because of its skewness and kurtosis but mainly because we can hardly expect participants to have a common understanding of “rarely, sometimes, and often.” The linguistic meaning of those frequency expressions is too vague. Consistent with this reasoning is Bocklisch et al. (2012), who showed that the positions and shape of participant response patterns varied considerably and that frequency expressions such as “rarely, sometimes, often” were hardly equidistantly distributed. Dichotomizing reduces this linguistic vagueness. We first dichotomized each item (0 indicates that the corresponding behavior was never observed; 1 indicates that this behavior was observed at least rarely; see also Zuber and Kaptein, 2014). Calculating the sum across all items then yielded the number of situations across which misconduct was observed (ranging from 0 to 19). This variable served as a criterion variable in the regression analyses.

The results of the bivariate correlations and hierarchical regression analyses are depicted in Table 6. The bivariate correlations between the ten dimensions composing the GECS and the deviant workplace behavior scale were all significant (*ps* < 0.01) and in expected directions (see Table 6, last column). Using regression analyses, we then tested how strongly sociodemographic variables (step 1), formal factors of ethics programs (step 2), compliance factors (step 3) and integrity factors (step 4) contribute to the variance in the outcome variable deviant workplace behavior. As scores for the GECS dimensions, we entered the factor scores of the PCA. As shown in Table 6, entering the formal factors (step 2) following the sociodemographic variables (step 1) increased the accounting for predicted variance from 3 to 6%. Entering the compliance factors of the GECS further increased the explained variance from 6 to 33%. However, entering the integrity factors again increased the explained variance from 33 to 48%. Thus, the results provide evidence for the incremental validity of the GECS over and above formal factors, but they also support the view that the integrity factors contribute to predicting observed misconduct even above the compliance factors.

**TABLE 6:** Results on hierarchical regressions and correlations with deviant workplace behavior (Sample B, Study 2).

	Step 1		Step 2		Step 3		Step 4		
	*β*	*SE*	*β*	*SE*	*β*	*SE*	*β*	*SE*	*r*
**Sociodemographics**									
Female	0.04	0.68	0.03	0.67	0.01	0.57	0.03	0.50	–0.07^*^
Age	–0.05	0.03	–0.06	0.03	0.03	0.03	0.05	0.02	–0.12^∗∗∗^
Job position	0.11^*^	0.31	0.13^**^	0.31	0.11^*^	0.26	0.10^*^	0.24	0.16^∗∗∗^
Years in company	–0.10	0.04	–0.08	0.04	–0.02	0.03	–0.02	0.03	–0.02
Size of the company	0.17^∗∗∗^	0.33	0.19^∗∗∗^	0.34	0.08	0.30	0.04	0.27	0.06
**Formal factors**									
Ethics code			–0.02	0.73	0.02	0.62	0.04	0.55	–0.02
Ethics training			0.03	0.84	–0.01	0.71	0.06	0.63	0.00
Telephone hotline			–0.22^∗∗∗^	0.77	–0.17^∗∗∗^	0.66	–0.12^∗∗^	0.58	–0.07^*^
Ethics office			0.10	0.93	0.10	0.79	0.07	0.70	0.07
Ethics control			–0.09	0.81	–0.03	0.72	–0.06	0.63	–0.02
Ethics report			0.10	1.01	0.10	0.86	0.04	0.77	0.11^∗∗^
**GECS – Compliance**									
Controlling					–0.13^∗∗^	0.30	–0.10^*^	0.27	–0.17^∗∗∗^
Sanctioning					0.02	0.31	0.03	0.29	–0.10^∗∗^
Rule clarity					–0.12^*^	0.29	–0.07	0.26	–0.28^∗∗∗^
Rule defectiveness					0.26^∗∗∗^	0.28	0.10^*^	0.27	0.43^∗∗∗^
Rule viability					0.31^∗∗∗^	0.29	0.10^*^	0.29	0.41^∗∗∗^
**GECS – Integrity**									
Accountability							–0.16^∗∗^	0.31	–0.29^∗∗∗^
Leader’s role modeling							0.01	0.30	–0.36^∗∗∗^
Pressure to compromise							0.40^∗∗∗^	0.30	0.58^∗∗∗^
Obedience							0.04	0.28	0.33^∗∗∗^
Ill-conceived goals							0.11^*^	0.28	0.36^∗∗∗^
*Adj. R*^2^ (Δ*R*^2^)	0.03		0.06 (0.03)		0.33 (0.27)		0.48 (0.15)	
*F* (Δ*F*)	3.71^∗∗^		3.51^∗∗∗^ (3.25^∗∗^)		14.57^∗∗∗^ (35.78^∗∗∗^)		20.51^∗∗∗^ (25.92^∗∗∗^)	

Dependent variable: sum of observed deviant workplace behaviors; ΔR^2^ and Δ*F* refer to a change in *R*^2^ and *F* statistics; *Max. VIF* refers to the largest variance inflation factor. ^*^p < 0.05, ^∗∗^*p* < 0.01, ^∗∗∗^*p* < 0.001.

Though not shown in the table, we also conducted an analysis using only the integrity factors (after entering the sociodemographic variables and ethics programs factors) to better compare the overall utility of the compliance versus integrity factors in predicting the criterion variable. This model explained 46% of the variance in deviant workplace behavior, while the model with the compliance factors, as mentioned above, explained 33% of the variance. These results further confirm that the utility of the compliance approach is relevant but somewhat weaker than that of an integrity- or value-oriented approach.

Overall, the final analysis revealed that 6 of 10 dimensions of the GECS were significant predictors of the extent of observed misconduct (*p*s < 0.05). The significant positive relations indicated that, as expected, participants reporting higher levels of rule defectiveness, rule viability, pressure to compromise, or ill-conceived goals were likely to observe more misconduct. The significant negative relations indicated that participants reporting higher levels of controlling or of accountability were likely to report less misconduct at work.

### Study 3 – Construct Validity

Data from Sample C (*N* = 991) were used to provide further evidence of the measure’s construct validity by comparing the GECS with other established measures of related constructs. We examined convergent validity by examining the relations between the composite measures of compliance and integrity factors and theoretically related constructs. As previous authors suggested that an integrity-based culture is more focused on enabling self-governance and personal commitment (e.g., Paine, 1994; Verhezen, 2010; Wieland et al., 2014), we expected integrity-based factors to correlate positively with *autonomous motivation* and negatively with *controlled motivation*. In contrast, given that a compliance-based culture is proposed to be more focused on controlling people, we hypothesized that compliance-based factors of the GECS would correlate positively with controlled but negatively with autonomous motivation. To assess those relations, we included a well-known *work motivation* measure (Gagné and Deci, 2005).

Furthermore, we included a measure of *duty orientation* reflecting people’s volitional obligation to perform organizational tasks, groups and missions (Hannah et al., 2014). This scale was originally developed in the context of active army soldiers but later also employed with government and corporate employees. This scale is interesting for us to examine construct validity, since it distinguishes between adherence to normative codes and rules (*duty to code*), the willingness to support and serve one’s group and its members (*duty to members*), and the willingness to support the purpose of the group and to make personal sacrifices (*duty to mission*). We expected both integrity and compliance-oriented factors of organizations to be positively related to duty orientation, since the purpose of both GECS components is ultimately to ensure employees’ consistency with organizational codes and standards. However, since compliance-oriented programs are more focused on compliance with rules, we also expected compliance factors to reveal somewhat higher positive correlations with duty to codes than would integrity factors. In contrast, as integrity-oriented programs are more focused on encouraging intrinsic, motivated commitment to standards, we expected integrity factors to reveal higher positive correlations with duty to members and mission than would compliance factors.

#### Measures

##### Work motivation

Beyond the GECS, a German version of the Multidimensional Work Motivation scale (Gagné et al., 2015) was administered. This measure assesses five distinct types of motivation along a continuum from extrinsic to intrinsic motivation. These types are combined into one category representing *controlled motivation* (11 items, e.g., “I am involved in my job, because others put pressure on me,” α = 0.85) and another one representing *autonomous motivation* (6 items, e.g., “I am involved in my job, because what I do in this job has a lot of personal meaning to me.”, α = 0.92). Items were rated on a 7-point scale from 1 (*strongly disagree*) to 7 (*strongly agree*).

##### Duty orientation

Duty orientation was assessed with the 12-item *Duty Orientation* scale (Hannah et al., 2014). Items were translated into German using the backtranslation method. Hannah and colleagues suggested that duty orientation is a higher-order factor that reflects three subordinate concepts, such as duty to codes, duty to members, and duty to mission. We therefore used the overall scale across all items (α = 0.86) and the subscales (duty to codes, α = 0.78; duty to members, α = 0.78; and duty to mission, α = 0.77). Sample items include “I do what is right always,” “I put the interest of my team ahead of my personal interest,” and “I do whatever it takes to not let the mission of my organization fail.” Items were rated on a 5-point scale (1 = *strongly disagree;* 5 = *strongly agree*).

#### Results

To examine convergent validity, we assessed the correlations between work motivation, duty orientation and the single and composite measures of compliance and integrity (Table 7). As shown, among the five integrity-based factors, leader’s role modeling, accountability and pressure to compromise mostly show a convergence with autonomous work motivation (*rs* > 0.24, *ps* < 0.001), while obedience and ill-conceived goals coincided with more-controlled motivation (*rs* > 0.21, *ps* < 0.001). Among the five compliance-oriented factors, we mainly found that rule clarity correlated positively with autonomous motivation (*r* = 0.25, *p* < 0.001) and that controlling (*r* = 0.22, *p* < 0.001) with correlated controlled motivation.

**TABLE 7:** Relation between GECS, work motivation and duty orientation (Sample C, Study 3).

**GECS**	***M* (*SD*)**	**Controlled motivation **	**Autonomous motivation**	**Duty orientation**	**Duty to codes**	**Duty to members**	**Duty to mission**
Controlling	3.19 (0.97)	0.22^∗∗∗^	0.10^∗∗^	0.15^∗∗∗^	0.16^∗∗∗^	0.11^∗∗∗^	0.12^∗∗∗^
Sanctioning	3.41 (0.90)	0.16^∗∗∗^	0.17^∗∗∗^	0.20^∗∗∗^	0.20^∗∗∗^	0.18^∗∗∗^	0.13^∗∗∗^
Rule clarity	3.89 (0.98)	0.18^∗∗∗^	0.25^∗∗∗^	0.22^∗∗∗^	0.21^∗∗∗^	0.24^∗∗∗^	0.11^∗∗∗^
Rule defectiveness	2.86 (1.05)	0.06	–0.15^∗∗∗^	–0.04	–0.09^∗∗^	–0.15^∗∗∗^	0.11^∗∗∗^
Rule viability	2.91 (1.05)	0.10^∗∗^	–0.16^∗∗∗^	–0.15^∗∗∗^	–0.16^∗∗∗^	–0.21^∗∗∗^	–0.02
Accountability	3.57 (0.82)	0.12^∗∗∗^	0.37^∗∗∗^	0.33^∗∗∗^	0.30^∗∗∗^	0.36^∗∗∗^	0.19^∗∗∗^
Leader’s role modeling	3.51 (1.02)	0.13^∗∗∗^	0.40^∗∗∗^	0.30^∗∗∗^	0.27^∗∗∗^	0.36^∗∗∗^	0.13^∗∗∗^
Pressure to compromise	2.28 (1.08)	0.11^∗∗∗^	–0.24^∗∗∗^	–0.13^∗∗∗^	–0.18^∗∗∗^	–0.27^∗∗∗^	0.08^*^
Obedience	2.95 (1.14)	0.21^∗∗∗^	–0.20^∗∗∗^	–0.05	–0.07^*^	–0.15^∗∗∗^	0.07^*^
Ill-conceived goals	2.54 (0.99)	0.30^∗∗∗^	–0.13^∗∗∗^	–0.02	–0.01	–0.20^∗∗∗^	0.13^∗∗∗^
Composite compliance factor	3.35 (0.61)	0.13^∗∗∗^	0.27^∗∗∗^	0.25^∗∗∗^	0.26^∗∗∗^	0.29^∗∗∗^	0.08^∗∗^
Composite integrity factor	3.46 (0.73)	–0.11^∗∗∗^	0.37^∗∗∗^	0.22^∗∗∗^	0.22^∗∗∗^	0.37^∗∗∗^	0.00

To calculate the composite compliance and integrity factors, items from the scales rule defectiveness, viability, pressure to compromise, obedience and ill-conceived goals were reversed. ^*^p < 0.05, ^∗∗^*p* < 0.01, ^∗∗∗^*p* < 0.00.

Note that for calculating the correlations between the composite measures of compliance, integrity and work motivation, the composite measures were constructed after reverse coding the negative items. Overall, supporting our expectation, the composite integrity factor correlated positively with autonomous work motivation (*r* = 0.37, *p* < 0.001) and negatively with controlled motivation (*r* = −0.11, *p* < 0.001). At least partially also supporting our hypothesis, the composite compliance factor correlated positively with controlled motivation (*r* = 0.13, *p* < 0.001) but also positively with autonomous motivation (*r* = 0.27) (even though to a lesser extent than with the composite integrity factor).

Furthermore, consistent with our expectations, both the composite integrity and compliance factor correlated positively with duty orientation (*rs* > 0.22, *ps* < 0.001), with leader’s role modeling and accountability revealing the largest correlations (*rs* > 0.30, *ps* < 0.001). Consistent with our more specific expectations, the compliance factors correlated somewhat more positively with duty to codes than did integrity factors, while integrity factors revealed higher correlations with duty to members than did compliance factors. Surprisingly, duty to mission revealed correlations only around zero.

Overall, the patterns of correlations in this study provided acceptable support for the convergent validity of the GECS. However, Table 7 also shows that the specific dimensions composing the compliance or integrity factor do correlate, though most of them in the expected direction, to various degrees with work motivation and duty orientation measures, suggesting that the compliance or integrity factors are not homogeneous factors but rather overlap somewhat.

## General Discussion

This research has taken first steps toward developing, refining and testing a German ethical culture measure. In doing so, we intended to advance the nomological network of corporate ethical culture by integrating the notion of compliance- and integrity-based ethics programs (with its focus on how to steer organizations) with the notion of ethical culture (with its focus on what factors inhibit or foster ethical behavior). In several studies involving more than 2000 participants, we presented first evidence for the factorial structure, reliability and validity of the instrument. Concerning the factor structure, fit indices based on data from various (sub)samples suggested the 10-factor solution as the best-fitting model among those tested. Considering the model with two overarching second-order factors (integrity and compliance) revealed acceptable or nearly acceptable fit indices. Item reliabilities of all subscales were good, ranging between 0.72 and 0.91. We found supporting convergent and divergent validity of the scale derived from CFA in most cases across the two samples.

Further supporting convergent validity of the scale, we found that compliance and integrity factors were in most cases predictably related to different forms of work motivation and duty orientation. The correlations were mostly modest but still significant. Composite compliance and integrity factors were mostly related in expected directions with controlled and autonomous work motivation and with variants of duty orientation (duty to codes and duty to members). The composite integrity factor was positively related to autonomous motivation but negatively to controlled motivation. The composite compliance factor, on the other hand, correlated positively with controlled motivation. However, somewhat unexpected was that the composite scale of compliance factors also correlated with perceived autonomous motivation (although to a lesser extent than did the composite integrity factor). This correlation may suggest that compliance factors can also be advantageous to autonomous motivation. As will be discussed later, this finding may provide an interesting avenue for future research.

Furthermore, the GECS revealed good predictive validity. Our studies showed that both compliance and integrity factors predicted significantly deviant workplace behavior beyond the formal factors of ethics programs. At the same time, the analyses of incremental validity stressed the added value of integrity factors in predicting deviant workplace behavior over and above compliance-base factors. Specifically, the integrity-based factors accounted for a 15% additional amount of variance beyond compliance factors. Consistent with previous studies (Treviño et al., 1999; Tyler and Blader, 2005), these results support the view that organizational strategies and factors relying on the monitoring of rule adherence may not be the only means of shaping people’s deviant behavior. Moreover, in the research reported here, the utility of a command and control approach overall appears to be weaker than that of an integrity- or value-oriented approach that attempts to influence ethical behavior by encouraging employee’s self-responsibility and moral motivation. Controlling for sociodemographic characteristics and ethics programs factors, the model including the compliance factors explained 33% of the variance of deviant workplace behavior, but the model including the integrity factors explained 46% of the variance.

Clearly, it would be useful to study the GECS together with other ethical culture approaches to examine the criteria and incremental validity of the GECS compared to those of other models. In our current research, we refrained from including other ethical culture questionnaires in our surveys simply to avoid too much burden from too lengthy questionnaires (e.g., the ethical culture questionnaire, the CEV, by Kaptein, 2008a, contains 58 items). Measurement economy must always be accounted for and carefully balanced out when running such studies. Nevertheless, a first rough comparison between the GECS and the CEV may be given building on Kaptein’s study (Kaptein, 2011), which included the nearly identical observed work deviant behavior scale as in our study. (The only differences were that Kaptein’s study included a list of 37 items of observed deviant workplace behavior and a general score calculated by averaging the scores across all items. Our study included a list of 19 behavioral items, and we calculated a general score by summing across all dichotomized items.) Conducting regression analyses, the author found that a model entering sociodemographic variables, formal factors of ethics programs and ethical climate (Victor and Cullen, 1987, 1988) accounted for 14% of the variance in observed unethical behavior. Upon entering the CEV, the exploratory power of the complete model increased to 31%. In our study, the analyses revealed that entering sociodemographic and formal factors of ethics programs accounted for 6% of the variance in observed unethical behavior. Upon entering the GECS with the 10 factors, the exploratory power of the complete model increased to 48%. This result is promising and indicates that, at least in this case, our measure appears to be superior in predicting observed unethical behavior.

This measure has several strengths. As a novelty, this measure integrates the notion of compliance- and integrity-based ethics program (with its focus on how to steer organizations) with the notion of ethical culture (with its focus on what factors inhibit or foster ethical behavior). To our knowledge, prior research on climate or culture has never considered this association, despite the prominence of the compliance and integrity distinction in theory and practice. At the same time, studies on compliance- and integrity-based ethics management have rarely conducted rigorous empirical research. We took the challenge to develop a measure to address the gap. Data gathered to test the scale were obtained across large samples, in two countries (Germany and Switzerland), and across a wide variety of organizations and jobs. This data collection is not taken for granted since many studies in this domain gather data within single organizations. Hence, the heterogeneity of our data allows some confidence to claim that the GECS is applicable across varying organizational contexts. Offering researchers a German scale will allow them to conduct stringent research in German-speaking countries on antecedents and consequences of compliance and integrity-oriented organizational cultures. In addition, such a measure allows organizations, risk managers and compliance officers to reflect the current state of corporate ethical culture within their own organizations, to design targeted interventions and to monitor organizational progress.

The present measure also represents an advancement to existing instruments in terms of including less-problematic item wording (to minimize social desirability, we avoided terms such as “moral”) and including current theoretical concepts. Drawing on important insights from behavioral ethics social psychology and organizational research, our measure incorporated dimensions such as ill-conceived goals, pressure to compromise and accountability. Furthermore, building on insights from practitioners, we incorporated dimensions such as rule defectiveness and rule viability. These concepts have thus far not been addressed in prior measures. In fact, it is interesting that nearly all of these factors were the most relevant predictors of deviant workplace behavior. Of course, we do not claim that our measure contains all relevant dimensions, but the findings indicate that our measure contains at least some important dimensions over and above previous measures.

Though the current measure is in German, we do not believe that the content is only applicable and relevant for the German-speaking world. In fact, compliance and integrity-based ethics programs are the leading approaches to ethics in today’s business world (e.g., OECD, 2009). Therefore, the application of the instrument in other countries would be not only very meaningful but also needed. Clearly, this process would also require testing and validation of the instrument in other languages and countries.

This research also has interesting implications for practice and future research. As mentioned above, the GECS offers practitioners a framework and measure to examine their state and progress within their own organization. In addition, our finding that integrity-based factors account for an additional amount of variance beyond compliance factors is relevant for practice, since most companies currently appear to focus primarily on improving control and command strategies in combating unethical behavior. Our results, however, strongly suggest that more attention should be focused on procedures that promote self-regulation and responsibility.

The GECS is also a basis for addressing empirical questions about the antecedents and consequences of compliance- and integrity-oriented cultures. Integrating the compliance and integrity distinction offers a unique opportunity to steer research in new directions. One fruitful direction for future research is certainly related to studying the various implicit or explicit assumptions about how compliance- and integrity-based cultures may be associated with employee motivation, attitudes and behavior (Paine, 1994; Verhezen, 2010). However, many of those assumptions still await empirical examination. In a recent theoretical paper, Stimmler and Tanner (2019) proposed that the potential implications of a compliance- or integrity-oriented culture may be better understood when considered in combination with perceptual, motivational, affective and behavioral processes at the individual level. For example, the regulatory focus theory (Higgins, 1997, 1998) can provide us with an interesting heuristic to deepen our understanding of what the psychological implications for the individual might be when an organization counts more on a compliance or integrity culture. With a regulatory focus, people can regulate their behavior via a prevention or promotion focus. Numerous empirical studies have shown that these different foci evoke different psychological processes (Higgins, 1997, 1998, 2001). We hypothesize that a compliance-oriented culture may be more likely to evoke a prevention focus, while an integrity-oriented culture may be more likely to support a promotion focus among employees. This avenue may be interesting for future research to examine these relations more thoroughly.

One specific direction for future research also relates to the, as mentioned above, somewhat unexpected finding that the composite compliance factor also correlated with autonomous work motivation. This finding could point to positive implications associated with compliance-oriented culture worth examining in more detail. Interestingly, empirical research in this domain is nearly absent, even though, for instance, the question about the possible implications of monitoring employees has been highly debated for many years (Frey, 1993; Ferrin et al., 2007). This literature primarily expect negative effects on employee work effort and intrinsic motivation. However, we speculate that, besides the potentially negative implications of a control- and command approach, such an approach may also be likely to provide employees with a comfortable feeling of certainty about the processes in the firm. An integrity-based culture, on the other hand, with its focus on encouraging self-governance, may create more uncertainty and uneasiness since it lacks explicit guidelines. It seems obvious that both compliance and integrity-oriented strategies are necessary for an effective ethical business culture. Nonetheless, our knowledge about the negative and positive implications associated with a compliance and an integrity-oriented culture and what combination or interplay may be most effective is severely limited.

Also interesting is the finding that leader’s role modeling did not emerge as a significant predictor of deviant workplace behavior, though past studies strongly emphasized the relevance of the “tone of the top” and leaders being role models to encourage similar behaviors among employees (e.g., Treviño et al., 1999; Kaptein, 2011). Leader’s role modeling, however, correlated highly positively with autonomous work motivation. This result suggests that role modeling is possibly more relevant for motivation than for behavior implications. Another possibility may be that leader’s role modeling is more likely to moderate the relationship between some other cultural dimensions and unethical behavior. It remains open for research to examine this issue in further detail.

Of course, this research also has limitations. The first one is associated with the fact that our analyses (CFA) revealed only moderate statistical support for dividing the single factors into composite compliance and integrity components. Although the GECS provides a first essential step in assessing compliance or integrity-based cultures, an important endeavor for further work is to further ameliorate the items and foci. In fact, whereas there is a fair consensus in the literature about what the governance strategies of a compliance-based program (such as control, monitoring, and sanctioning) are, there are still divers opinions about exactly what procedures constitute an integrity-based program. Thus far, we suggested accountability and leader’s role modeling as crucial building blocks of an integrity culture. Future discussions and work will probably reveal further strategies that may then be incorporated into the GECS.

Another limitation refers to the fact that some expectations about the relations between compliance and integrity factors were not confirmed. For example, in contrast to our expectations, integrity factors did not correlate with duty to mission (neither did the compliance factors). This finding might be caused by the fact that the duty orientation scale was originally developed in the context of army soldiers (Hannah et al., 2014). According to Hannah et al. (2014) and taking a closer look at the duty to mission items, this subconcept is about individual’s willingness to make personal sacrifices and accept personal risks to achieve the goals of the group. We suppose that in the context of “normal” organizational life, such expectations of employees are too unrealistic and therefore likely to result in correlations around zero.

Finally, a limitation of this research may be the possibility of a common method bias. Recall that in study 2, the same respondents answered the GECS and deviant workplace behavior questions. One strength of our studies is that data were collected from heterogeneous samples, including employees and managers across various organizations. This variety has allowed us to examine the robustness of the proposed structure across heterogeneous organizational contexts. Although we administered observed rather than self-reported misconduct and used different response formats to assess the predictor and criterion variables to reduce this problem, we cannot completely exclude the possibility that the associations between the predictor and criterion measures may be biased. Thus, future research should attempt to collaborate with single organizations and to assess the predictor and criterion variable for different employees from the same working groups within an organization.

Overall, we believe that the GECS provides a first measure to build upon to advance research in German-speaking countries, to provide a basis for empirically examining antecedents and the short- and long-term consequences of compliance- and integrity-based cultures, and to improve our understanding of the influences that may hinder or facilitate the effectiveness of such procedures. In this vein, we strongly believe that the GECS can fuel future research and make contributions to the field of organizational ethics.

## Data Availability

The datasets generated for this study are available on request to the corresponding author.

## Ethics Statement

The studies were carried out in accordance with the ethical guidelines of the Zeppelin University. The online studies were approved by the Ethics Committee of the Zeppelin University and the Department of Psychology at the University of Zurich. The studies were carried out in accordance with the APA ethical guidelines, guidelines of the Swiss Psychological Society, and the European Commission (Horizon 2020). All participants indicated affirmative consent by clicking the button, “I have read the information and I agree with them.”

## Author Contributions

CT and KG designed the studies and collected the data. NW and KG did the analyses. All authors contributed to the writing and agreed to be accountable for all aspects of the work.

## Conflict of Interest Statement

The authors declare that the research was conducted in the absence of any commercial or financial relationships that could be construed as a potential conflict of interest.
