# Supplementary material for: The German Ethical Culture Scale (GECS): Development and First Construct Testing
Source: Front Psychol. 2019 Jul 16;10:1667. doi: 10.3389/fpsyg.2019.01667 (PMC6646868; doi:10.3389/fpsyg.2019.01667)
Supplement: Supplementary file 1 [file Table_1.DOCX]

Supplementary Material

Table: Items of the German Ethical Culture Scale (GECS)

| **Subscales/Items** | |  | | |
| --- | --- | --- | --- | --- |
| **German original** [**English translation**] | | | | **Abbreviation** |
| **Kontrolle** [**Controlling**] | | |  | |
|  | Wenn meine Führungskraft etwas Unerlaubtes tut, wird es jemand in der Organisation herausfinden. [If my manager does something that’s not allowed, someone in the organization will find out about it.] | | | Controlling1 |
|  | Wenn einE MitarbeiterIn etwas Unerlaubtes tut, wird es jemand in der Organisation herausfinden. [If a colleague does something unauthorized, someone in the organization will find out about it.] | | | Controlling2 |
|  | In meinem Arbeitsumfeld werden Kontrollen durchgeführt, um Regelverstöße und Fehlverhalten aufzudecken. [In my work environment, measures are carried out to detect rule violations and misconduct.] | | | Controlling3 |
| **Bestrafung** [**Sanctioning**] | | |  | |
|  | In meinem Arbeitsumfeld werden Beschäftigte, bei denen menschliches Fehlverhalten entdeckt wurde, vom Management diszipliniert, unabhängig von ihrer Stellung in der Organisation. [In my work environment, employees who are found to have committed misconduct are disciplined by management, regardless of their position within the organization.] | | | Sanctioning1 |
|  | In meinem Arbeitsumfeld werden Personen, die sich unredlich verhalten, maßgeregelt. [In my work environment, people who engage in dishonest behavior are disciplined.] | | | Sanctioning2 |
|  | In meinem Arbeitsumfeld sind vor allem Personen erfolgreich, die sich an die Normen, Regeln und Richtlinien der Organisation halten. [In my work environment, the most successful people are those who follow the norms, rules, and guidelines of the organization.] | | | Sanctioning3 |
| **Klarheit** [**Rule Clarity**] | | |  | |
|  | Meine Organisation macht mir ausreichend klar, welche Verhaltensweisen richtig oder falsch sind. [My organization makes it sufficiently clear to me which behaviors are right or wrong.] | | | RuleClarity1 |
|  | Meine Organisation macht mir ausreichend klar, wie ich mich in der Organisation anderen gegenüber angemessen verhalten soll. [My organization makes it sufficiently clear to me how to behave appropriately toward others.] | | | RuleClarity2 |
|  | Meine Organisation macht mir ausreichend klar, wie ich verantwortungsvoll mit externen Personen und Organisationen umgehen soll. [My organization makes it sufficiently clear to me how to deal responsibly with external people and organizations.] | | | RuleClarity3 |
| **Unvollständigkeit** [**Rule Defectiveness**] | | |  | |
|  | In den Organisationsregeln werden manche Dinge bewusst nicht oder unscharf geregelt. [Some of the organization’s rules are intentionally not or vaguely defined.] | | | RuleDefectiveness1 |
|  | In meiner Organisation gibt es in den Organisationsregeln Grauzonen. [There are gray zones in the organization’s code of conduct.] | | | RuleDefectiveness2 |
|  | In meiner Organisation gibt es moralisch schwierige Herausforderungen, zu denen keine klaren Vorgaben existieren. [In my organization, there are morally difficult challenges for which no clear guidelines are given.] | | | RuleDefectiveness3 |
| **Brauchbarkeit** [**Rule Viability**] | | |  | |
|  | Die Verhaltensregeln der Organisation erhöhen den bürokratischen Aufwand. [The organization’s code of conduct increases the burden of bureaucratic labor.] | | | RuleViability1 |
|  | Die Verhaltensregeln der Organisation machen alles kompliziert. [The organization’s code of conduct makes everything complicated.] | | | RuleViability2 |
|  | Die Verhaltensregeln der Organisation gehen an den Anforderungen aus der Praxis vorbei. [The organization’s code of conduct is not meeting practical needs.] | | | RuleViability3 |
| **Rechenschaftspflicht** [**Accountability**] | | |  | |
|  | In meinem Arbeitsumfeld übernehmen die Beschäftigten die Verantwortung für die erfolgreiche Umsetzung einer Initiative. [In my work environment, employees are responsible for the successful implementation of an initiative.] | | | Accountability1 |
|  | In meinem Arbeitsumfeld verantworten die Beschäftigten die guten aber auch schlechten Konsequenzen ihrer Arbeit. [In my work environment, employees are accountable for the good and the bad consequences of their work.] | | | Accountability2 |
|  | In meiner Arbeit übernehmen die Menschen freiwillig Verantwortung für anfallende Aufgaben. [In my workplace, people willingly undertake responsibility for the tasks assigned to them.] | | | Accountability3 |
|  | In meiner Arbeit gehen die Beschäftigten mit Arbeitsressourcen (z.B. Zeit, Geld, Kontakte) verantwortlich um. [In my workplace, employees handle work resources (time, money, contacts) responsibly.] | | | Accountability4 |
|  | In meiner Arbeit spielt es auch eine Rolle wie man Ziele erreicht hat. [In my workplace, how goals are achieved also plays a role.] | | | Accountability5 |
|  | In meiner Arbeit wissen alle, wer für welche Informationen oder Aufgaben Ansprechpartner ist. [In my workplace, everyone knows who is in charge for which information or tasks.] | | | Accountability6 |
| **Führungskraft als Vorbild** [**Leader’s Role Modeling**] | | |  | |
|  | Meine direkte Führungskraft trifft faire und ausgewogene Entscheidungen. [My direct supervisor makes fair and balanced decisions.] | | | RoleModeling1 |
|  | Meine direkte Führungskraft verhält sich den MitarbeiterInnen gegenüber wertschätzend und respektvoll. [My direct supervisor behaves appreciatively and respectfully toward colleagues.] | | | RoleModeling2 |
|  | Meine direkte Führungskraft ist ein gutes Vorbild für Integrität. [My direct supervisor is a good model of integrity.] | | | RoleModeling3 |
|  | Das obere Management ist ein gutes Vorbild für Integrität. [The top management is a good model of integrity.] | | | RoleModeling4 |
|  | Für das obere Management ist soziale Verantwortung nicht nur ein leeres Wort. [For the top management, social responsibility is not an empty phrase.] | | | RoleModeling5 |
| **Kompromissdruck** [**Pressure to Compromise**] | | |  | |
|  | In meinem Arbeitsumfeld werde ich manchmal aufgefordert Dinge zu tun, die in Konflikt zu meinem Gewissen stehen. [In my work environment, I am sometimes asked to do things that are in conflict with my conscience.] | | | PressureToCompromise1 |
|  | Um in meiner Organisation erfolgreich zu sein, muss ich manchmal meine eigenen Normen und Werte opfern. [To be successful in my organization, I sometimes have to sacrifice my own norms and values.] | | | PressureToCompromise2 |
|  | In meiner Organisation wurde ich schon angehalten, Regeln zu brechen. [In my organization, I have already been encouraged to break the rules.] | | | PressureToCompromise3 |
|  | In meiner Arbeitstätigkeit befinde ich mich häufig in einem Wertekonflikt. [In my work activities, I often find myself in a conflict of values.] | | | PressureToCompromise4 |
| **Gehorsamkeit** [**Obedience**] | | |  | |
|  | Von mir wird erwartet zu tun, was man mir sagt. [I am expected to do what I am told.] | | | Obedience1 |
|  | Von mir wird erwartet, dass ich ohne Widerrede meiner Führungskraft gehorche. [I am expected to obey my manager without argument.] | | | Obedience2 |
|  | In meiner Arbeit wird Widerspruch nicht toleriert. [Opposition is not tolerated in my workplace.] | | | Obedience3 |
| **Schlecht durchdachte Ziele** [**Ill-Conceived Goals**] | | |  | |
|  | An meinem Arbeitsplatz geht es nur um quantifizierbare Leistungsziele. [My workplace is all about quantifiable performance targets.] | | | IllConceivedGoals1 |
|  | An meinem Arbeitsplatz verfolgen die Beschäftigten nur ökonomische Ziele. [At my workplace, the employees have only economic goals.] | | | IllConceivedGoals2 |
|  | An meinem Arbeitsplatz mache ich nur Karriere, wenn ich die anderen übertrumpfe. [In my workplace, I can only make a career by outperforming others.] | | | IllConceivedGoals3 |
|  | An meinem Arbeitsplatz werde ich in erster Linie danach beurteilt, ob ich mehr erreicht habe als meine ArbeitskollegInnen. [In my workplace, I am evaluated first and foremost on whether I have achieved more than my colleagues.] | | | IllConceivedGoals4 |
|  |  | | |  |
